# Bacterial Cellulose: Production, Modification and Perspectives in Biomedical Applications

**DOI:** 10.3390/nano9101352

**Published:** 2019-09-20

**Authors:** Selestina Gorgieva, Janja Trček

**Affiliations:** 1 Faculty of Mechanical Engineering, Institute of Engineering Materials and Design, University of Maribor, 2000 Maribor, Slovenia; 2 Faculty of Electrical Engineering and Computer Science, Institute of Automation, University of Maribor, 2000 Maribor, Slovenia; 3 Faculty of Natural Sciences and Mathematics, Department of Biology, University of Maribor, 2000 Maribor, Slovenia; janja.trcek@um.si; 4 Faculty of Chemistry and Chemical Engineering, University of Maribor, 2000 Maribor, Slovenia

**Keywords:** bacterial cellulose, carbon source, in situ modification, ex situ modification, biomedical applications

## Abstract

Bacterial cellulose (BC) is ultrafine, nanofibrillar material with an exclusive combination of properties such as high crystallinity (84%–89%) and polymerization degree, high surface area (high aspect ratio of fibers with diameter 20–100 nm), high flexibility and tensile strength (Young modulus of 15–18 GPa), high water-holding capacity (over 100 times of its own weight), etc. Due to high purity, i.e., absence of lignin and hemicellulose, BC is considered as a non-cytotoxic, non-genotoxic and highly biocompatible material, attracting interest in diverse areas with hallmarks in medicine. The presented review summarizes the microbial aspects of BC production (bacterial strains, carbon sources and media) and versatile in situ and ex situ methods applied in BC modification, especially towards bionic design for applications in regenerative medicine, from wound healing and artificial skin, blood vessels, coverings in nerve surgery, dura mater prosthesis, arterial stent coating, cartilage and bone repair implants, etc. The paper concludes with challenges and perspectives in light of further translation in highly valuable medical products.

## 1. Introduction

Cellulose is one of the most abundant biopolymers on Earth, and is mainly of plant, wood and bacterial origin. The cellulose of bacterial origin exhibits the highest purity and has thus attracted the interest of many researchers and industrial sectors. Generally, it consists of randomly assembled, <100 nm wide ribbon-shaped fibrils, composed of 7–8 nm-wide elementary nanofibrils aggregated in bundles. As such, it delivers a combination of exclusive properties, such as flexibility, high water holding capacity, hydrophilicity, crystallinity, mouldability in different shapes, elevated purity with absence of lignin and hemicellulose and biomimetic three-dimensional (3D) network as a hallmark. Because of these features, this type of cellulose attracts interest for different medical applications such as the engineering of artificial skin (particularly in recuperation of burned skin), artificial blood vessels, topical covering for severe wounds, coverings in nerve surgery, dura mater prosthesis, arterial stent coating, wound dressings, hemostatic material, electronic platforms, implants for cartilage and bone repair etc.

**Figure 1:**
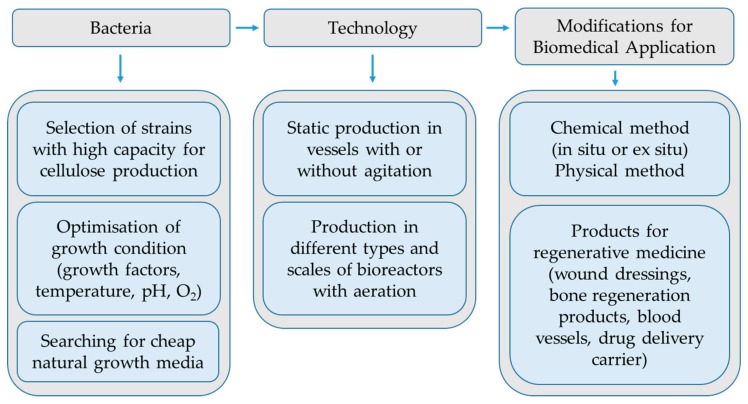
Scheme presenting the most important aspects which have to be considered for bacterial cellulose (BC)-production with biomedical application.

For efficient bacterial cellulose (BC) production we need an efficient and stabile bacterial strain with demands for growing that are not too expensive and with ability of being easily scaled up to industrial settings. The produced cellulose is generally easily separated from growth medium and further on modified using different approaches for various medically relevant applications. All these aspects (Figure 1) will be discussed in this review paper.

## 2. Bacteria Have High Capacity for Cellulose Production

BC is nanofibrillar, extracellular polysaccharide produced by diverse bacteria when they are growing statically, but also when bacteria are submerged in liquid and cultured by shaking. Bacteria produce BC in media with different carbon sources, although the efficiency of BC production differs substantially among various growth substrates. The substrate supplies energy to bacterial metabolism during the exhaustive energy-consuming pathway of cellulose synthesis. Theoretically, every carbon block which the bacterial cell metabolizes into glucose, can be used for cellulose production [1,2].

The capacity of BC production is widespread among bacteria, but the most prominent and well-known BC-producer is species *Komagataeibacter xylinus*, which belongs to the group of acetic acid bacteria (AAB). AAB are strictly aerobic Gram-negative bacteria classified into *α-Proteobacteria* [3,4,5]. The species has been for many years known as *Acetobacter xylinum*, but has been later classified into *Gluconacetobacter xylinus* and due to further taxonomic changes finally reclassified into *Komagataeibacter xylinus*. *K. xylinus* is not the only species among AAB with an immense potential for BC production, since also other species, such as *Komagataeibacter hansenii*, *Komagataeibacter medellinensis*, *Komagataeibacter nataicola*, *Komagataeibacter oboediens*, *Komagataeibacter rhaeticus*, *Komagataeibacter saccharivorans* and *Komagataeibacter pomaceti* have been characterized as strong cellulose producers [4,6,7,8,9]. An important aspect of using AAB for cellulose production is their characteristic of being food-grade or GRAS bacteria (generally recognized as safe). 

BC is synthesized in bacterial membrane from nucleotide-activated glucose [10]. Bacteria then channel BC through pores of cell membrane as fibrils composed of D-glucose units which are linked with β-1,4-glycosidic bonds. The chain is linear and extruded from the cell. Then the lateral and unidirectional aligned chains form intra- and inter-chain hydrogen bonding through all available hydroxyl groups. In this way the chains merge into insoluble nanofibrils of up to 25 nm in width and 1 to 9 μm in length which represents 2000 to 18,000 glucose residues [11]. These nanofibrils further aggregate into <100 nm wide ribbon-shaped fibrils what delivers a combination of exclusive properties to BC such as high water holding capacity, hydrophilicity, crystallinity and mouldability. Although almost all hydroxyl groups of the cellulose polymer are occupied with hydrogen bonds, one end of each cellulose polymer carries an unmodified C4-hydroxyl group and the opposite end a free C1-hydroxyl group, both of them representing possible sites for chemical modifications of cellulose [12].

Synthesis of nucleotide-activated glucose takes place in bacterial cytoplasm. If the starting substrate is glucose, the uridine diphosphate (UDP)-glucose is produced in three steps: phosphorylation of glucose by glucokinase, isomerization of glucose-6-phosphate into glucose-1-phosphate by phosphoglucomutase and synthesis of UDP-glucose by uridylyltransferase (UTP)-glucose-1-phosphate. Finally, cellulose synthase transfers glucosyl residues from UDP-glucose to the nascent β-D-1,4-glucan chain. Cellulose synthase is a membrane-embedded glycosyltransferase composed of two or three subunits [13]. The catalytic subunit of cellulose synthase is a major determinant of chemical and physical properties of BC, meaning that different bacterial species are able to generate cellulose with different lengths [14].

Comparison of AAB genomes revealed that AAB can possess more operons for cellulose production, moreover, the composition of operons differs from each other [4,15]. These differences very likely influence cellulose synthesis, cellulose transport to the cell surface and/or assembly of fibrils into ribbons [1]. 

The BC is not the only extracellular polysaccharide secreted by AAB. The other two well-known extracellular polysaccharides, acetan and levan are, however, water soluble [4,16,17]. Interestingly, acetan was first described in species *Komagataeibacter xylinus*. In contract to cellulose, acetan is branched acidic heteropolysaccharide [18]. Ishida et al. [19] identified lower cellulose production in a mutant not producing acetan. However, the cellulose production could be recovered by addition of acetan into the medium, meaning that the synthesis of both polymers is not connected at the genetic level. 

## 3. Different Carbon Sources Used for Bacterial Cellulose (BC) Production

The production of BC is extremely expensive, which is mainly a consequence of high costs of synthetic media for its production. The most well-known complex synthetic medium for growing cellulose producing AAB is Hestrin–Schramm medium (HS), composed of 2% (w/v) glucose, 0.5% (w/v) peptone, 0.5% (w/v) yeast extract, 0.27% (w/v) Na_2_HPO_4_ and 1.15 g/L citric acid [20]. During BC production, other by-products, such as gluconic and other acids are formed, that can decrease the BC yield [8]. The composition of HS medium can be further optimized for the highest cellulose yield by replacing glucose with other carbon sources, such as maltose, fructose, cellobiose, mannitol, xylose, sucrose, galactose etc. In most cases glucose turned out to be the best energy source for bacteria, besides, glucose can be directly used as precursor for the assembly of glucose units into cellulose. Wang et al. [2] have recently reported that fructose had in their microbial process higher cellulose yield in comparison to other carbon sources, also to glucose. The process for BC production can be further optimized by adding buffers into medium for keeping pH at optimal value for growing bacterial strains [6]. 

**Figure 2:**
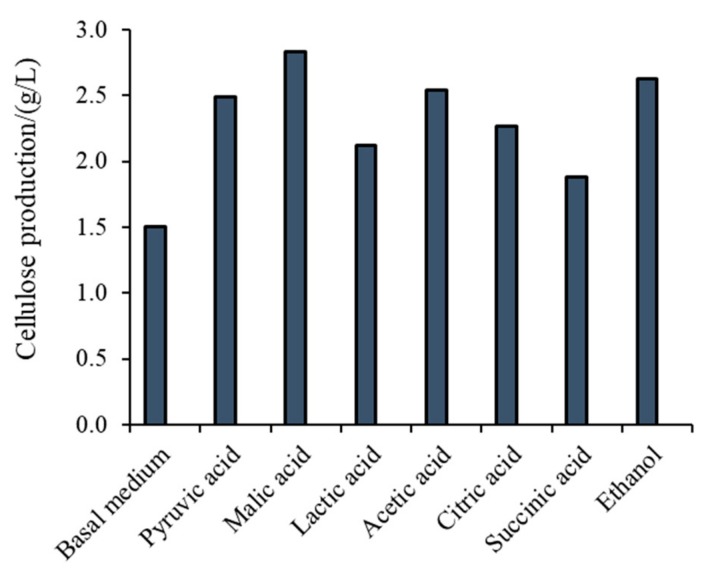
Influence of different organic acids and ethanol on cellulose yield. Reproduced from [21] with permission from Research & Reviews: Journal of Microbiology and Biotechnology, 2016.

To reduce the costs for BC production, the alternative natural carbon sources are utilized, such as waste substrate from different sectors of the food industry, sugar cane molasses etc. The BC yield can be improved also by addition of additives into growth medium such as glycerol, agar, xanthan, sodium alginate, ethanol (Figure 2), carboxymethyl cellulose (CMC), etc. Naritomi et al. [21] reported on enhanced cellulose yield during continuous BC production with *K. xylinus* subsp. *sucrofermentans* BPR3OOlA using fructose medium supplemented with 0.1 wt% of ethanol. The production of cellulose in a static culture with strain *K. xylinus* DA increased about 4-fold as a result of adding 2 wt% acetic acid in glucose medium [22]. Lu et al. [23] reported enhanced BC production with *K. xylinus* in chemically defined medium under static cultivation by the addition of pyruvic acid, malic acid, lactic acid, acetic acid, citric acid, succinic acid, and ethanol (Figure 2) in concentrations 0.15%, 0.1%, 0.3%, 0.4%, 0.1%, 0.2%, 4%, respectively. Li et al. [24] improved cellulose production with the strain *K. hansenii* M2010332 by the addition of ethanol and sodium citrate. Lu et al. [25] reported that the addition of 1% of methanol, 0.5% ethylene glycol, 0.5% of n-propanol, 3% of glycerol, 0.5% of n-butanol and 4% of mannitol produced 21.8%, 24.1%, 13.4%, 27.4%, 56% and 47.3% higher yield of cellulose by culturing strain *K. xylinus* 186 statically in glucose medium. The experiments of Matsuoka et al. [26] showed that the addition of lactate and methionine in fructose medium improved cellulose production with *K. xylinus* subsp. sucrofermentas BPR200. However, the BC yield reached 90% of that obtained in corn steep liquor. There is also a report on improved BC production with *K. xylinus* ATCC 10,245 by adding vitamin C in growth medium [27].

The production of BC in synthetic media with different carbon sources and growth factors, which are usually added as yeast extract and peptone, is expensive. The researchers are thus searching for inexpensive raw material containing high levels of sugars as substrates for BC production. To this aim several raw materials have been analyzed for BC production, such as tobacco waste extract [22], sugar beet molasses, cheese whey media [23], distillery effluent [24], corn steep liquor [25], fruit juice [26], corn stalks [28], litchi extract [29], beverage industrial waste [30], corncob acid hydrolysate [31] and waste beer yeast [32]. Another possible natural growth medium would be waste material from wine production. According to recent reports [33], 1.17 kg of grapes are used to produce 750 mL wine, and after the grapes are squeezed, about 20% of that weight remains in the form of grape skins, seeds and stems, counting for ~12 million tons each year. This substrate contains soluble carbohydrates (white grapes), fibers, acids, salts, and phenolic compounds (red grapes) [34] and as such it is often considered as a convenient source of carbon for microbial processes. Moreover, grape waste as carbon source in BC production may contribute to reduce winery residuals, reduce BC production costs, offering new ways to diversify BC production by taking into account also the environmental aspect by diminishing waste products in nature. 

The carbon source used for growing BC-producers affects BC properties: water holding capacity, surface area, porosity, polymerization degree, molecular weight, crystallinity index (67%−96%), mean crystallite size (5.7−6.4 nm), intrinsic viscosity, oxygen and water vapor transmission rates, mechanical properties, etc. Molina-Ramírez et al. [35] reported improved BC yield by addition of ethanol and acetic acid in growth medium, however, the crystallinity index, the degree of polymerization and maximum rate of degradation temperatures decreased by 9.2%, 36%, and 4.96%, respectively, by the addition of ethanol and by 7.2%, 27%, and 4.21%, respectively, by the addition of acetic acid. The crystallinity index of BC produced in the presence of ascorbic acid also decreased with remarkable change in d-spacing [27]. However, a recent study of Wang et al. [2] reported similar morphology and microfibrils of BCs from different carbon sources, meaning that these characteristics have to be checked for each bacterial strain before starting BC production at large scale.

**Figure 3:**
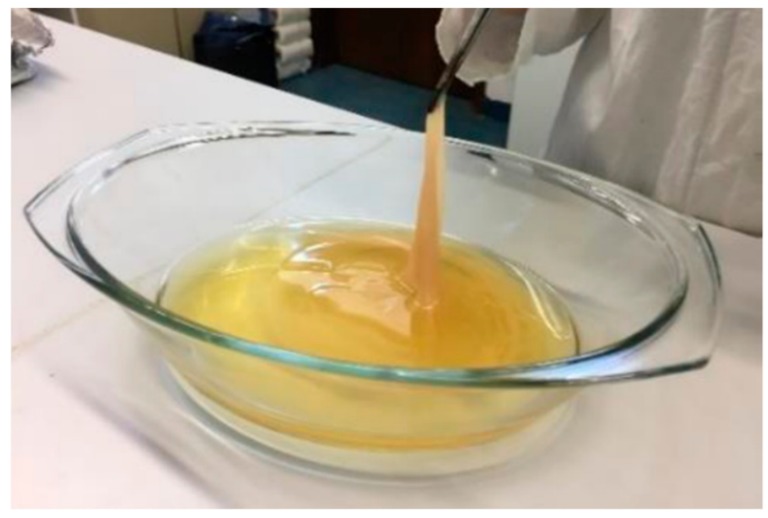
Static production of cellulose by *Komagataeibacter maltaceti* 1529^T^ on complex microbiological medium.

The production of BC can be simply performed in vessels with large surface area which support direct supply of oxygen and assembly of large cellulose sheets (Figure 3). To improve the efficiency of BC production and to produce cellulose of desired characteristics, different technological approaches can be used (Table 1). 

## 4. BC Modifications with Medical Relevance

**Figure 4:**
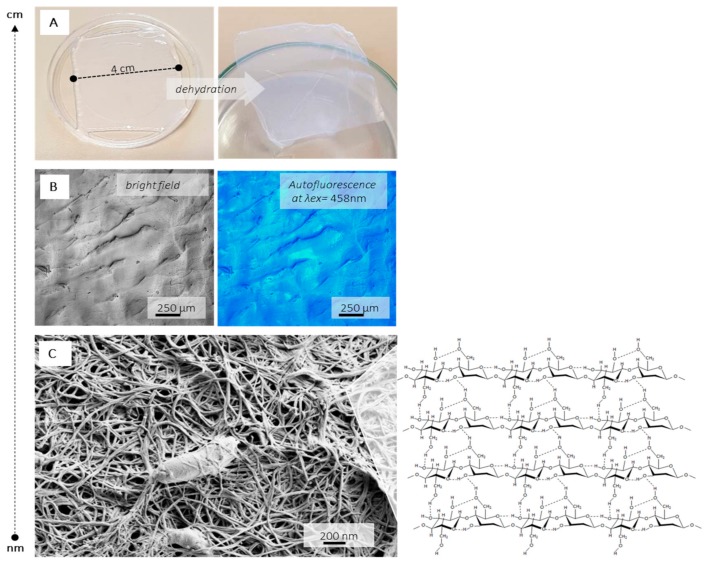
Different length-scale presentation of BC: (**A**) photographs of wet (left) and dry (right) BC membrane, (**B**) confocal fluorescent microscopy (CFM) image obtained under argon laser excitation at 458 nm from bright field and fluorescence channel, utilizing the cellulose autofluorescence and (**C**) high magnification scanning electron microscopy (SEM) image presenting entrapped *K. xylinus* bacteria and cellulose backbone insert.

3D structuring of BC within a translucent, gelatinous, interwoven, nano-fibrous network of linear polysaccharide polymers is formed at static conditions, as displayed within Figure 4. In comparison with vegetal cellulose sources, BC demonstrate remarkable mechanical properties, such as flexibility [42] and soft-tissue resembling stress-strain behavior [43], as well as a high level of crystallinity and water-holding capacity. BC is a very pure material where common cellulose associates, i.e., lignin and hemicellulose, are absent. As such, is considered a non-cytotoxic, non-genotoxic and highly biocompatible material.

However, BC lacks appropriate functionalities to trigger the initial cell attachment and control over the porosity, and it has very slow degradation, etc. To overcome this, BC has been modified by chemical (modification of chemical structure and functionalities) and physical means (change in porosity, crystallinity and fiber density) by applying versatile in situ and ex situ methods. In situ modifications are performed by the variation of culture media, carbon source and addition of other materials, while ex situ modifications are carried out by chemical and physical treatment of formed BC.

Chemical modification rely on inherent chemical reactivity due to the presence of hydroxyl groups, allowing reaction not only at heterogeneous, but also under homogeneous conditions. When compared with plant cellulose, the BC was found to be more reactive towards cynoethylation and carboxymethylation [44]. The homogeneous reaction including dissolving of BC with acetic anhydride and further iodination also reveals the highest reactivity of BC, yet, such a type of modification destroys the nanofibrillar structure.

Variation of water content within BC largely influences its viscoelastic and electrochemical properties. Due to increased resistance of BC to electron transfer, it becomes stiff at 50%–80% of water [45]. Such a finding was particularly important in wound dressing applications, where moisture content is an imperative. Addition of water-soluble polymers, such as CMC, methylcellulose (MC), and poly(vinyl alcohol) (PVA), was found to influence the water content of never dried and re-swollen BC [46]. On the other hand, Bottan et al. [47] introduced the guided assembly-based biolitography as technique to change the BC surface topography what is related to migratory patterns and alignments of human dermal cells, the fibroblasts and keratinocytes.

Some of modifications and resulting properties of BC are summarized within Table 2.

### 4.1. In Situ Modifications 

**Figure 5:**
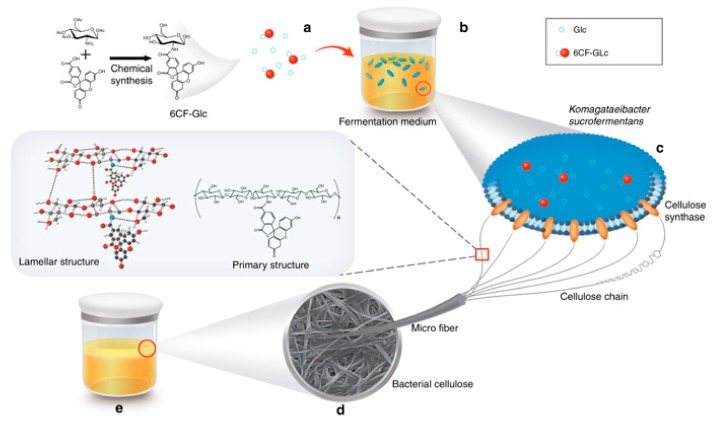
Synthesis of 6CF-BC by in situ microbial fermentation method, using glucose (Glc) modified with 6CF as a carbon source for *K. sucrofermentans* fermentation. (**a**) Glc and 6CF-Glc molecules; (**b**) microorganism fermentation; (**c**) the synthesis of 6CF-BC fibers through *K. sucrofermentans*, (**d**) microstructure of 6CF-BC; (**e**) the 6CF-BC pellicle obtained through microorganism fermentation. Reproduced from [65], with permission from Nature Communications, 2019.

Several studies identify in situ modifications as straightforward approach for introduction of particular functionality to BC by addition of reinforcement material (chitosan, gelatin, poly-3-hydroxybutirate, nanomaterials, clays, silica) to the bacterial culture medium, mostly at the beginning of BC production. The great advantage of such a process is encaging materials that become part of the fibrils, thus enhancing BC by altering mainly the physical–mechanical properties of BC fibrils. Moreover, new functionalities also can be introduced. Recent work of Gao et al. [65] propose in situ introduction of glucose being pre-modified with carboxyfluorescein (6CF), which supplements the BC with green fluorescence signal based on ultraviolet (UV) spectroscopy and confocal microscopy detection as presented by Figure 5.

For application in regenerative medicine and tissue engineering, the BC modification emphasis is on extracellular matrix (ECM) recapitulation [66], yet approaches are application-dependent and vastly diverse. Bone tissue engineering requires the presence of a bioactive component like hydroxyapatite Ca_5_(PO_4_)_3_OH (HAp) and tricalcium phosphate (TCP) Ca_3_(PO_4_)_2_ and several research works report on their inclusion within BC culture medium, resulting in BC/hyaluronic acid (HA) composite with high bone regeneration capacity. BC/HA composite prepared in the process of the cellulose biosynthesis with the introduction of aqueous HAp suspension, allows simultaneous formation of microfibrillar stripes and partial texturing of HA crystals onto them [67]. The addition of CMC in growth media modify medium’s viscosity and thus positively impacts assembling of calcium-deficient Hap powders formation in post synthetic stage, while not affecting the composite biocompatibility [68]. For vascular tissue engineering applications, the heparin-modified BC was produced by adding heparin to growth media of BC-producers, thus resulting in anticoagulant sulfate groups-enriched BC-heparin hybrid [69]. Other study introduces chitosan to BC trough in situ approach, being further ex situ modified with heparin, ending up with BC/chitosan/heparin composites with antimicrobial and anticoagulant properties [70]. For tissue-regeneration procedures, where porosity is an essential property, the paraffin microspheres were added to BC culture medium, resulting in microporous BC for bone regeneration [71], urinary conduit formation [72], etc. For wound-healing and temporary artificial skin applications, the BC culturing media is supplemented with glucose, dextrin [73], potato starch, cotton gauze, *Aloe vera*, which allows processing of composites, where only morphologies and physical properties are altered and not the chemical composition of BC itself. Addition of deacetylated chitin nanocrystals to BC culture media resulted in composite with bactericidal activity [74], while CMC addition introduced the surface charge, effective for further conjugation to affibody ligands applicable in tubular bio-filtration of blood proteins [66]. 

Apart from published studies, the critical limitation of the in situ modification approach presents incorporation of reinforcement materials that also have antibacterial activity against BC strains, the insolubility of various materials in culture media, high surface tension towards hydrophobic materials, the lack of structure control of BC nanofibers, and introduction of particles with low suspension stability within BC growing media, etc. 

**Figure 6:**
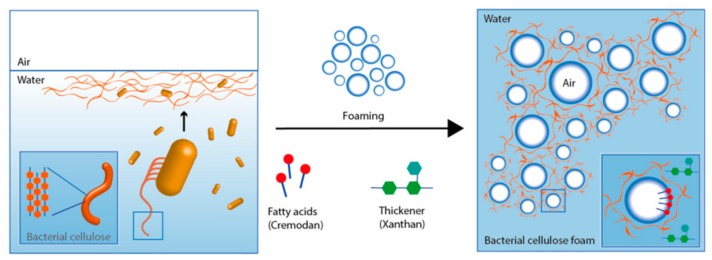
Schematic presentation of the BC foam formation process by *K. xylinus* suspension foaming and stabilization by Cremodan and xanthan as a thickener. Reproduced from [75], with permission from npj Biofilms and Microbiomes, 2018.

In situ modification of BC porosity is not affected by the aforementioned limitations and several studies demonstrate facile procedure for pore size manipulation. As shown by Lu et al. [60], the addition of potato starch to culture medium increases BC viscosity by interrupting BC assembly during static culture and thus creating more free spaces within the fibrous network. Further culturing of muscle cells onto loose surface of produced scaffolds results in new biomaterials for hollow organ reconstruction. The procedure for processing of macro-porous and foam-like BC was recently reported by Rühs et al. [75]; they cultured *K. xylinus* in mannitol-based media by foaming and then stabilized the product with surfactant Cremodan and viscosified with xanthan to prevent water drainage (Figure 6). 

### 4.2. Ex Situ Modifications 

**Figure 7:**
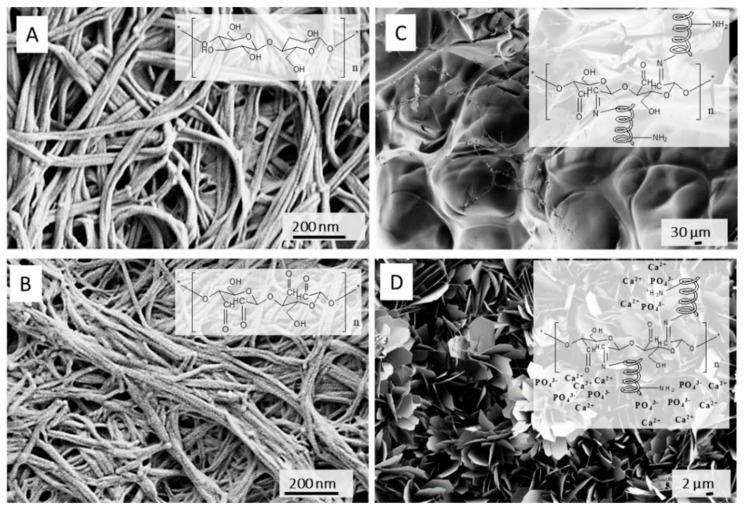
Scanning electron microscopy images of (**A**) native and post-synthetically modified BC; (**B**) oxidation with NaIO_4_; (**C**) further coupling with gelatin (GEL), carbodiimide crosslinking and freeze-thawing; (**D**) in situ mineralization by incubation in (10× concentrated) simulated body fluid medium. Adapted from [92], with permission from Nanomaterials, 2019.

Ex situ modifications are either chemical (e.g. periodate oxidation and grafting [76] or crosslinking reactions) or physical (physical absorption from solutions or particle suspensions, the homogenization or dissolving of BC mixing with additive material [77]). The BC is compounded with bioactive materials for applications such as tracking of tumor cells behavior [78], enhancement of osteoblasts cell growth in bone regeneration, fibroblast/endothelial cells guide in wound healing, etc. For the replacement of small blood vessels and improvement of the adhesion of human endothelial cells, the BC surface was modified with Arg-Gly-Asp (RGD) tripeptide, directly [79] or indirectly through xyloglucan-Gly-Arg-Gly-As-Ser (XG-GRGDS) conjugates [80]. For blood clothes control, the isolated BC from nata di coco was compounded with different fractions of kaolin [81]. To mimic the glycosaminoglycans of cartilage tissue, the surface charge was added to BC by means of chemical phosphorylation and sulfatation [82]. Incorporation of N-containing groups on BC was succeed by nitrogen plasma treatment, which also improved its porosity and enhanced the attachment of neuroblastoma (N1E-115) and human dermal microvascular endothelium (HMEC-1) cells. For application as a wound dressing, the BC was immersed into chitosan solution, forming BC/chitosan composite with high water-retention capacity [83]. For cardiovascular soft tissue replacement applications, the BC suspension was mixed with PVA, which improves the final mechanical performance [84]. Soaking of BC in silk fibroin solution results in nanocomposite with enhanced cell permissiveness, keeping the non-cytotoxicity and non-genotoxicity as in native BC [76]. For introducing antimicrobial activity against *Escherichia coli*, *Staphylococcus aureus* and *Candida albicans* while keeping biocompatibility of BC towards human embryonic kidney cells, the sodium alginate solution with silver sulfadiazine was mixed with BC slurry and further cross-linked with CaCl_2_ [85]. Different type of nanoparticles were simultaneously formed and introduced into BC- the antimicrobial ZnO [86] and Ag nanoparticles [87], where BC was initially impregnated with zinc acetate and silver nitrate, respectively. The bone morphogenetic protein-2 was introduced into BC to promote the bone regeneration [88]. Other reported BC modifications are gentamicin-, RGD-grafted BC [89], the gelatin-grafted BC using procyanidin [90], phosphorylation [91], etc. The periodate oxidation was used for region-selective oxidation of BC and further coupling with gelatin biopolymer (Figure 7). Such composite demonstrate improved physiological degradation (compared to non-degradable, native BC) as well as capacity for accommodation of flake-like apatite minerals in short-term incubation within supersaturated simulated body fluid (SBF) [92].

## 5. BC in Regenerative Medicine

**Figure 8:**
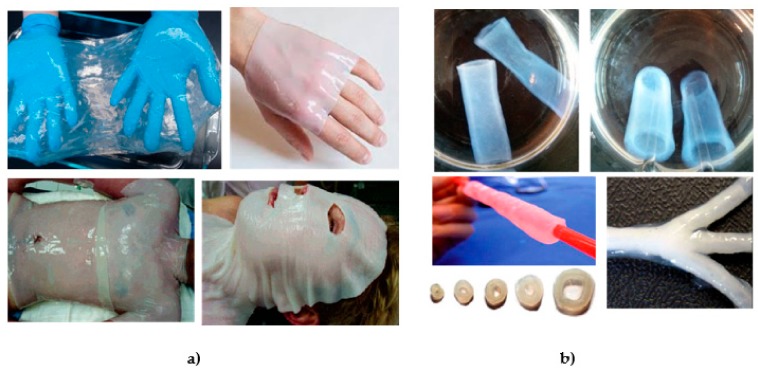
(**a**) BC dressings as produced and when applied on wounded torso, face and hand. Reproduced from [106], with permission from Biomacromolecules, 2007; (**b**) vascular graft and blood vessel tubes with different sizes and shape, produced by fermentation onto a branched silicone tube. Reproduced from [107,108,109], with permission from Frontiers, 2016, European Polymer Journal, 2014 and Biotechnology and Bioengeneering, 2007, respectively.

Nanocellulose materials attract significant attention in biomedical materials research [93,94,95] devoted to tissue engineering [96], cell [97] and gene therapy [98], diagnostic [99] and controlled delivery [100], mainly related to their nano-features and properties arising from them. For BC, there is also ultra-high purity and net-like morphology similar to (human) collagen as a biomimetic feature, which facilitates applications such as artificial skin (Figure 8a), vascular grafts (Figure 8b), tissue-engineering scaffolds, dental implants, medical pads, artificial bone and cartilage, delivery of drugs, proteins and hormones [101]. Several commercially available products are available on market, applied during skin transplantation, second and third degree ulcer treatment, decubitus, substitution of dura mater in bran, recovery of periodontal tissues, etc. The biocompatibility assessment of BC implant, by means of chronic inflammation, foreign body responses, cell ingrowth, and angiogenesis evidence no macroscopic signs of inflammation around the implants, absence of fibrotic capsule or giant cells and fibroblasts infiltration without chronic inflammatory reaction [102].

BC efficiency in wound healing generally relies on effective cohesion with wound boundaries, preservation of a moist environment (important for re-epithelization) combined with exudates retention capacity, high mechanical strength at wet state, liquid/gasses permeability, very low risk for irritation due to its ultra-high purity, and ease of wound inspection due to its transparency [103,104,105], etc. In case of chronic wound treatment with BC-based wound dressing materials, the reduction of proteolytic enzymes activity, cytokines and production of reactive oxygen species are reported.

Even though BC possess many insintric features that encourage its use in wound dressing, its commercial dissemination is not exhaustively exploited yet [104]. The first BC-based commercial medical product was Biofill^®^, a thin BC film with a water content of 8.5%. Material is used as a temporary skin substitute and wound dressing in treatment of basal cell carcinoma, severe burns, dermal abrasions, chronic ulcers as well as at donor and receptor sites in skin grafts. Pain relief, close adhesion to the wound bed, spontaneous detachment following reepithelization and reduced treatment times as well as costs, yet limited elasticity, when applied in areas of great mobility, are related to this product [110]. Membracell^®^ is also a temporary skin substitute used in treatment of burns and ulcers, sim providing pain relief, reduced infection, faster healing, etc. Bionext^®^ and Xcell^®^ are wound-dressing materials with similar outcomes [111]. Nanoderm™ is wound treatment product for acute and chronic wounds, allowing a barrier to infections while allowing gaseous exchange, exudate evaporation, and pain alleviation, acting as a regenerative tissue scaffold to affect fibroblast, endothelial and keratinocyte function, enhancing granulation tissue formation and epithelization [112]. The Cellumed^®^ product is used in veterinary medicine for treatment of large surface wounds on horses and [113].

Further incorporation of inorganic (Ag [114], ZnO [114], CuO [115] and TiO_2_ particles [116]) and organic antimicrobial agents (lysozyme [117], ε-poly lysine [53], nisin [118] garlics’ allicin [119]), evoke their effectiveness against several bacterial strains (*Staphylococcus aureus* and *Escherichia coli*), as well as fungal strains (*Aspergilus niger* and *Candida albicans*). Abdominal hernia treatment is another application of BC as a dressing material, where better absorption in native tissue with less risk of mesh-related infections, impact and hypersensitivity at the implant site were reported [120]. 

A recent strategy in treatment of skin injuries is incorporation of mesenchymal stem cells, the adult pluripotent cells that can differentiate more than two cell times [121]. Loh et al. [122] seeded the human epidermal keratinocytes and dermal fibroblasts onto BC/acrylic acid hydrogel and further transferred them to a wound, reporting that the procedure accelerated the healing process. 

Porosity, mouldability, foldability, hemocompatibility and good mechanical properties are attributes which position BC also in blood vessel replacement applications [123]. Especially in replacement of small blood vessels (<5 mm) as alternative to thorax or legs-harvested vessels or synthetic Dacron, extended Polytetrafluoroethylene (ePTFE) and polyurethane (PU) materials [124]. Control over porosity is prime requirement, as proliferation and migration of endothelial cells within the membrane is essential when semi-synthetic products are considered. Composite with graphene oxide [125], functionalization with chimeric proteins (conjugates of cellulose binding module and RGD adhesion peptides) [126], blending with PVA polymer [127] are among reported studies where coagulation issues and hemocompatibility are toughly investigated. 

A commercial product used in the area of guided tissue and bone regeneration is Gengiflex®, the two-layer membrane comprised of native and alkali-modified BC, used for treating the osseous deficiency surround TiAl_6_V_4_ (IMZ) dental implant with simultaneous restoration of the aesthetic and mouth function [128]. This product was shown to support recovery of periodontal tissue by reduced inflammatory response, requiring fewer surgical steps. Saska et al. [129] reported a combination of glycine-modified BC and type I collagen with high alkaline phosphatase (ALP) activity for bone tissue regeneration. For same application, the hydroxyapatite-coated BC was investigated by Ahn et al. and new bone formation within rat calvarian defect model in 8 weeks study was defined as highly promising outcome [130]. Complexation capacity of phosphorylated BC towards calcium was utilized in study of augmentation of mineralization yields and migration of bone-forming osteoprogenitor cells [131,132].

**Figure 9:**
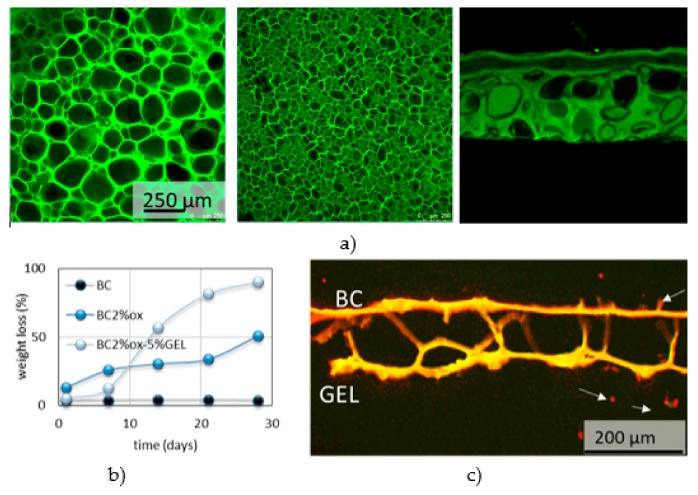
(**a**) Fluorescent microscopy images of top, bottom and cross-section aspect of BC-gelatin composite membranes; (**b**) their degradation kinetic; (**c**) barrier effect towards MRC-5 cells. Adapted from [92], with permission from Nanomaterials, 2019.

In a recent study, Gorgieva et al. [92] combined BC membrane with gelatin utilizing successive periodate oxidation and a freeze-thawing/carbodiimide crosslinking procedure, which forms µ-porous composite membrane. Acting as a barrier for fibroblast penetration, the membrane did not evoke any cytotoxic effects toward human fibroblast (MRC-5) cells, while the same preferentially attached on a gelatin porous site (Figure 9).

In neural tissue engineering, Innala et al. [133] reported that BC adapts to the SH-SY5Y neuroblastoma cells, which adhered, proliferated and differentiated towards mature neurons as measured by electrophysiological data. A study generated a 3D model that can be used for developing in vitro disease models. For example, combining this scaffold with human-induced pluripotent stem cells that have been derived from diseased patients, the 3D model can be used for detailed investigations of neurodegenerative disease mechanisms and in the search for new therapeutics [133].

The absence of suitable polymers and proteins, and the presence of low endotoxin units (according to the U.S. Food and Drug Administration (FDA) legislation), further expands the BC application portfolio towards drug-delivery applications [134,135], especially to tuning the drug release kinetic and optimization of drug concentration. Amin et al. [136] reported pH sensitive hydrogel formulations of BC with polyacrylic acid and bovine serum albumin as a model drug. Another study investigate BC membranes with added photosensitizer, chloroaluminum phthalocyanine for photodynamic therapy in skin cancer treatment [137].

Other cellulosic fibers (nitrocellulose in particular) have a long history as anchoring substrate for antibody conjugation in diagnostic assays [138], where also the BC appear as suitable candidate. Major effort in this area is given to processing on homogenous, 3D films in order to increase the quantity of antibodies to be further anchored. BC combination with PVA was investigated as artificial cornea [61] and aortic heart valve leaflet [139]. Tronser et al. [140] identify BC as convenient material enabling for long-term maintenance of mouse embryonic stem cells, simultaneously facilitating their culturing and handling.

## 6. Perspectives and Challenges for BC

BC offers an inestimable platform for development within the biomedical field, especially towards high-tech products, from nursing and diagnostic to theranostic and highly demanding regenerative, tissue-engineering products. However, more effort needs to be made in initial production steps and the fact that AAB productivity towards BC production varies strongly among different species and strains, as well as the carbon source, opens room for additional basic research input in this area. Traditional carbon sources in BC production are glucose, fructose and glycerol, which significantly increases expenses, presenting ~30% of total BC production costs. The industrial wastes or by-products have recently been proposed as cheap local sources for BC production. Some examples are corn steep liquor (CSL)-fructose medium, which is a fully enriched medium with minerals, inositol, nicotinic acid, thiamine and pantothenic acid. Date syrup and molasses are other alternatives, being highly competitive with traditional Hestrin–Schramm and Yamanaka media in BC production. Alternative carbon sources, (i.e., what straw, fruit juices, rotten fruit, waste from cotton textiles, dairy industries, biodiesel industries are already suggested) may potentially enlarge, speed up and cheapen BC production. As such, they have not been fully explored to the stage of semi-final, biomedical products. This in turn will seek more facile, cost-effective and industry-translatable modifications beyond standard post-synthetic oxidation and grafting pathways. Potential “housing” of selected and suitable biopolymers or particulates within BC during the synthetic procedure while keeping in mind no restricted BC production is one way to tackle the problem.

## Figures and Tables

**Table 1:** The most common methods for bacterial cellulose (BC) production.

Method for BC Production	Basic Characteristics of The Process and The Cellulose
Static production [36]	Most commonly used method at the lab scale. Duration of the process is up to two weeks. Cellulose is in the form of hydrogel sheet.
Production in shaking culture [37,38]	Increased delivery of oxygen to bacteria. Might result in reduced genetic stability of bacteria and lower BC production. Production of cellulose of different particle sizes and various shapes (mainly of spherical structure). Suitable for economic scale production.
Production in airlift bioreactor [38,39]	Efficient oxygen supply with low power supply. Cellulose produced in pellet.
Production in rotating disc bioreactors [40]	Production of homogenous cellulose. Cellulose yield is compared to the static process.
Production in trickling bed reactor [41]	Provides high oxygen concentration and low shear force. Produce BC in form of irregular sheets.

**Table 2:** Modifications of BC and resulting properties.

Modification	Application	Resulting Properties
BC nanocrystals/Regenerated Chitin fibers (BCNC/RC) [48]	Suture biomaterials	Biocompatible surgical sutures increasing strength of BCNC/RC filaments; Enzymatic degradation possible; Degradation rate can be tuned by varying concentration of BCNCs in the yarn; Chitin can promote cell proliferation (in vivo).
BC with modified topography [47]	Wound dressing	Improved cell alignment; Promotion of fibroblast infiltration and new collagen deposition in the wound bed.
Vaccarin impregnated on BC [49]		Neovascularization; Stratified squamous epithelium; Dense new- born subcutaneous tissue formation of collagen fibers and hyperplastic fibrous connective tissue.
2,2,6,6-Tetramethylpiperidinyloxy (TEMPO)-Oxidized BC with Ag nanoparticles [50]		Antimicrobial activity; Ag^+^ release with a rate of 12.2%/day at 37 °C in 3 days; Biocompatible.
BC/ZnO nanocomposite [51]		Antimicrobial activity against *Escherichia coli, Pseudomonas aeruginosa, Staphylococcus aureus* and *Citrobacter freundii;* Significant healing of 66% after 15 days related to day 0.
BC/TiO_2_ nanocomposite [52]		Antimicrobial activity against *Escherichia coli* and *Staphylococcus aureus.*
BC/ε -poly-L-Lysine (ε-PLL) nanocomposite [53]		Antimicrobial activity (broad-spectrum) without affecting the beneficial structural and mechanical properties; Modification with non-toxic biopolymer ε-PLL inhibited growth of *S. epidermidis* on the membranes but did not affect the cytocompatibility to cultured human fibroblast.
BC/Ag nanoparticle composite [54,55]		Environmentally benign and facile approach; Sustained release of Ag; Prolonged antibacterial performance against *Staphylococcus aureus.*
Silymarin (SMN)-zein nanoparticle/BC nanocomposite [56]		Change of wettability and swelling; Antioxidant and antibacterial activity; Air-dried SMN-zein/BC nanocomposite slow down the lipid oxidation.
BC/Octenidin/Poloxamer hybrid system [57]	Drug deliveryWound treatment	Long term controlled release of octenidine; Improved mechanical and antimicrobial properties; Ready-to-use system with Poloxamer-loaded BC for advanced treatment of infected wounds; Non toxicity in test with shell-less hen’s egg model.
BC/CMC/Methotrexate [58]		Impact of DS-CMC on methotrexate loading; Topical treatment of psoriasis; Decrease of the elastic modulus as the degree of substitution (DS) of CMC increased;
BC/PHEMA Hydrogel matrice [59]	Biomedical application	New modification: in situ ultraviolet (UV) radical polymerization; Tensile strength increased; Nontoxic; Rat mesenchymal stem cells (rMSCs) proliferation; Tissue replacement and wound healing.
BC with tuned porosity [60]	Tissue engineering	Higher pore size than native BC to allow muscle cell ingrowth; Small decrease in mechanical strength.
BC/PVA composite [61] BC/Hyaluronic acid (HA) [62]	Artificial cornea	Higher visible light transmittance than plain BC.
BC/urinary bladder matrix [63]	Retinal pigment epithelium	Higher adhesion and proliferation of retinal pigment epithelium cells than uncoated BC; Closer recapitulation of the *in vivo* cell phenotype than uncoated BC.
BC/iron oxide nanoparticles [64]	Blood vessels	Introduction of magnetic domains; Young's modulus correspond to values for blood vessels.
